# DTranNER: biomedical named entity recognition with deep learning-based label-label transition model

**DOI:** 10.1186/s12859-020-3393-1

**Published:** 2020-02-11

**Authors:** S. K. Hong, Jae-Gil Lee

**Affiliations:** 1 Graduate School of Knowledge Service Engineering, KAIST, 291 Daehak-ro, Yuseong-gu, Daejeon, 34141 South Korea; 2 Department of Industrial & Systems Engineering, KAIST, 291 Daehak-ro, Yuseong-gu, Daejeon, 34141 South Korea

**Keywords:** Bioinformatics, Data mining, Named entity recognition, Neural network

## Abstract

**Background:**

Biomedical named-entity recognition (BioNER) is widely modeled with conditional random fields (CRF) by regarding it as a sequence labeling problem. The CRF-based methods yield structured outputs of labels by imposing connectivity between the labels. Recent studies for BioNER have reported state-of-the-art performance by combining deep learning-based models (e.g., bidirectional Long Short-Term Memory) and CRF. The deep learning-based models in the CRF-based methods are dedicated to estimating individual labels, whereas the relationships between connected labels are described as static numbers; thereby, it is not allowed to timely reflect the context in generating the most plausible label-label transitions for a given input sentence. Regardless, correctly segmenting entity mentions in biomedical texts is challenging because the biomedical terms are often descriptive and long compared with general terms. Therefore, limiting the label-label transitions as static numbers is a bottleneck in the performance improvement of BioNER.

**Results:**

We introduce DTranNER, a novel CRF-based framework incorporating a deep learning-based label-label transition model into BioNER. DTranNER uses two separate deep learning-based networks: Unary-Network and Pairwise-Network. The former is to model the input for determining individual labels, and the latter is to explore the context of the input for describing the label-label transitions. We performed experiments on five benchmark BioNER corpora. Compared with current state-of-the-art methods, DTranNER achieves the best F1-score of 84.56% beyond 84.40% on the BioCreative II gene mention (BC2GM) corpus, the best F1-score of 91.99% beyond 91.41% on the BioCreative IV chemical and drug (BC4CHEMD) corpus, the best F1-score of 94.16% beyond 93.44% on the chemical NER, the best F1-score of 87.22% beyond 86.56% on the disease NER of the BioCreative V chemical disease relation (BC5CDR) corpus, and a near-best F1-score of 88.62% on the NCBI-Disease corpus.

**Conclusions:**

Our results indicate that the incorporation of the deep learning-based label-label transition model provides distinctive contextual clues to enhance BioNER over the static transition model. We demonstrate that the proposed framework enables the dynamic transition model to adaptively explore the contextual relations between adjacent labels in a fine-grained way. We expect that our study can be a stepping stone for further prosperity of biomedical literature mining.

## Introduction

Biomedical named-entity recognition (BioNER) automatically identifies specific mentions of interest such as chemicals, diseases, drugs, genes, DNAs, proteins, viruses etc. in biomedical literature. As the fundamental step for various downstream linguistic tasks, e.g., adverse drug event extraction [1], bacteria biotope task [2], drug-drug interaction [3], and protein-protein interaction detection [4], the performance of BioNER is crucial in the overall biomedical knowledge discovery process [2].

BioNER operates by predicting a class label for each token across biomedical literature. It is typically considered as a sequence labeling problem and is thus widely modeled by a first-order linear-chain conditional random field (CRF) [5, 6]. CRF yields chain-structured label sequences by collectively assessing possible label-label transition relations between words over the entire input sequence.

In recent years, deep learning (briefly, DL) has become prevalent across various machine learning-based natural language processing (NLP) tasks since neural network-based learning systems can effectively identify prominent features in a data-driven way, replacing task-specific feature engineering based on high-level domain knowledge [7, 8]. For NER tasks, recent methods [9–14] have reported state-of-the-art performance by introducing a bidirectional long short-term memory (BiLSTM) into CRF. Accordingly, the combination of BiLSTM and CRF has been widely considered as a standard architecture for various sequence labeling problems.

The combined models (i.e., BiLSTM-CRFs) for NER typically consist of two major components: a token-level BiLSTM and a real-valued transition matrix. The BiLSTM is dedicated to estimate the best-suited label on each token, while the transition matrix is solely responsible for describing the transition compatibility between all possible pairs of labels on neighboring tokens; in detail, the numerical score at the *i*th row and *j*th column of a transition matrix represents the transition compatibility from the *i*th label to the *j*th label. Note that the transition matrix is once established by being suited to the statistics of given training data via its parameter learning and is frozen afterward. As a result, the transition matrix cannot provide the contextualized compatibility for the relationship of neighboring labels in a fine-grained way.

Accordingly, we contend that solely relying on the static transition matrix is not enough to explain the ever-changing label-label transition relations in BioNER, since biomedical entities are frequently descriptive, long or even contain conjunctions [15], e.g., “normal thymic epithelial cells,” “peripheral sensor neuropathy,” and “central nervous system and cardiac toxicity.” As a result, the boundaries of entity-mentions in biomedical texts are often too ambiguous to accurately segment them. Therefore, we argue that exploiting contextual information to describe label-label transition relations is important to facilitate the accurate identification of biomedical entities. Recently, Lin et al. [16] studied that explicitly modeling relations between parts in a structured model is applicable to semantic image segmentation, whereas it has been rarely studied in recent DL-based NLP methods.

To this end, we propose a novel framework, called *Dynamic Transition for NER (DTranNER)*, to incorporate a DL-based model, which adaptively identify label-label transition relations to further improve the accuracy of BioNER. Overall, DTranNER makes use of two separate DL-based models: Unary-Network and Pairwise-Network. The addition of Pairwise-Network makes it possible to assess the transition compatibility between adjacent labels by exploring the context of an input sentence. Meanwhile, as another DL-based model, Unary-Network is used for individual labeling as in previous works. After all, Unary-Network and Pairwise-Network are arranged to yield agreed label sequences via this novel framework.

Because DTranNER is orthogonal to a DL-based model, any type of DL-based models such as attention [17] or transformer [18] can be employed to play the role of Unary-Network or Pairwise-Network. In this study, we conduct experiments using a BiLSTM as the underlying DL networks since it has been widely adopted in various sequence labeling problems so far.

We evaluated DTranNER by comparing with current state-of-the-art NER methods on five benchmark BioNER corpora to investigate the effectiveness of the DL-based label-label transition model. The results show that DTranNER outperformed the existing best performer on four out of five corpora and showed comparable accuracy to the existing best performer on one remaining corpus, thereby demonstrating the excellent performance of DTranNER.

## Background

### Problem definition: biomedical named entity recognition (BioNER)

An instance of a BioNER corpus consists of an input token sequence *x*=*x*_1_,…,*x*_*N*_ and its associated output label sequence *y*=*y*_1_,…,*y*_*N*_. We use the IOBES tagging scheme by which tokens are annotated with one of “I,” “O,” “B,” “E,” or “S” labels. In the case of an entity spanning over multiple tokens, “B” is tagged to the token to indicate the beginning of the entity, “I” stands for “Inside,” and “E” indicates the ending token of the entity. For the case of an entity of a single token, the “S” label is tagged to it. The “O” label stands for “Outside,” which means that the token is not part of any named entity. To indicate the type of entities, one of the type tags, such as “Chemical,” “Disease,” “Gene,” or “Protein,” is additionally concatenated to each IOBES tag.

### Linear-chain conditional random field (CRF)

As a class of discriminative probabilistic graphical models, a linear-chain conditional random field (CRF) describes the joint probability *P*(**y**|**x**) of the entire structured labels **y** with respect to the structure of an undirected graph, given a set of inputs **x**. CRF is widely used in various sequence labeling problems as well as BioNER by imposing the first-order Markov property on the output sequence labeling. There are two types—unary and pairwise—of elementary feature functions to organize an output label sequence. The unary feature functions are dedicated to estimating the suitability of candidate labels at each individual position, whereas the pairwise feature functions are designed to assess possible pairwise labels on two connected positions. Summing up, when an input sequence **x** of length *N* is given, the conditional distribution *P*(**y**|**x**) is represented as a product of position-dependent unary and pairwise feature functions; thus, it is formulated as in the following equation:

1 $$\begin{array}{*{20}l} P(\mathbf{y}|\mathbf{x}) = \frac{1}{Z(\mathbf{x})}\exp(\sum\limits_{i=1}^{N}{\sum\limits_{j}^{J}{\lambda_{j}^{s}s_{j}(y_{i},\mathbf{x},i)}} \\ +\sum\limits_{i=2}^{N}\sum\limits_{k}^{K}\lambda_{k}^{t}t_{k}(y_{i-1},y_{i},\mathbf{x},i)), \end{array} $$

where *s*_*k*_(*y*_*i*_,**x**,*i*) denotes a member of the unary feature functions (i.e., *s*∈*S*) at the position *i*, and *t*(*y*_*i*−1_,*y*_*i*_,**x**,*i*) indicates a member of the pairwise feature functions (i.e., *t*∈*T*) at two consecutive positions *i-1* and *i*. Traditionally, the unary and pairwise feature functions are manually designed to facilitate accurate sequence labeling, and they are usually real-valued binary indicators representing either true or false. The weights (i.e., *λ*^*s*^∈*θ*_*s*_ and *λ*^*t*^∈*θ*_*t*_) associated to the feature functions are trainable parameters. *Z*(**x**) is the partition function as a normalization constant over all possible label assignments.

### Bidirectional long short-term memory (BiLSTM)

Long short-term memory (LSTM) [19] is a specific variant of recurrent neural networks to mitigate the problem of vanishing and exploding gradients in modeling long-term dependencies of a sequence. LSTM is suited for modeling sequential data with recurrent connections of hidden states *H*={*h*_1_,*h*_2_,…,*h*_*N*_} and have become ubiquitous in a wide range of NLP tasks. At every time step, LSTM yields a current hidden state $\overrightarrow {h_{t}}$ and internally updates a current cell state $\overrightarrow {c_{t}}$ based on $\overrightarrow {h_{t-1}}$ and $\overrightarrow {c_{t-1}}$ calculated in the previous time step.

Given that LSTM is limited to using past context in the forward direction, a bidirectional LSTM (BiLSTM) is employed to exploit future context as well as past context. BiLSTM processes an input sequence in both forward and backward directions with two separate LSTMs. That is, the hidden states from both directional LSTMs are concatenated to make final output vectors $h_{t}=\{\overrightarrow {h_{t}}\oplus \overleftarrow {h_{t}}\}$.

### Merger of BiLSTM and CRF: BiLSTM-CRF

BiLSTM-CRF has been widely employed in recent neural network-based NER studies [9–14, 20, 21] for sequence labeling. The architecture of BiLSTM-CRF is typically comprised of four layers: a token-embedding layer, a token-level BiLSTM layer, a binding layer, and a CRF layer. We denote an input token sequence of length *N* by **x**={*x*_1_,⋯,*x*_*N*_} and the corresponding output label sequence by **y**={*y*_1_,⋯,*y*_*N*_}. First, the token-embedding layer encodes input tokens into its fixed-dimensional vectors as *e*_1_,*e*_2_,…,*e*_*N*_. Next, the BiLSTM layer takes the token-embedding vectors as the inputs to generate the hidden-state vectors *h*_1_,*h*_2_,…,*h*_*N*_. Before being fed to the CRF layer, the hidden-state vectors are transformed to the score vectors *U*_1_,*U*_2_,…,*U*_*N*_ with *L*-dimensionality, where *L* denotes the number of labels, via the binding layer so as to match the number of labels. The score vector contains the confidence values for possible labels on its corresponding token position. Namely, the stack from the token-embedding layer to the binding layer can be considered to play the role of the unary feature functions (i.e., *s*∈*S*) in Eq. 1. Besides, a real-valued transition matrix, denoted as *A*, accounts for all the label-label transition relations; it is likewise regarded to play the role of the pairwise feature functions (i.e., *t*∈*T*) in Eq. 1. Eventually, BiLSTM-CRF calculates the likelihood for a label sequence **y** given an input token sequence **x** via the following equation:

2 $$ P_{u}(\mathbf{y}|\mathbf{x}) = \frac{1}{Z}\exp{(\sum\limits_{i=1}^{N}U_{i}(y_{i})+\sum\limits_{i=2}^{N}A_{i-1,i})},   $$

where *U*_*i*_(*y*_*i*_) denotes the unary score for assigning the label *y*_*i*_ on the *i*th token, *A*_*i,j*_ corresponds to the real-valued pairwise transition compatibility from *i*th label to *j*th label, and $Z\,=\,\sum \nolimits _{\mathbf {y}}{\exp {(\sum \nolimits _{i=1}^{N}U_{i}(y_{i})+\sum \nolimits _{i=2}^{N}A_{i-1,i})}}$.

## Related work

Recent state-of-the-art CRF-based NER studies [9–14, 20–22] have demonstrated the effectiveness of data-driven representation learning (i.e., DL) under CRF. We discuss several CRF-based methods for NER in terms of two kinds of feature functions: unary and pairwise feature functions. We also introduce BioBERT that showed the state-of-the-art performance in BioNER.

- Lample et al. [9] proposed to bring BiLSTM into CRF for NER in general news domain. The model uses two BiLSTMs: one for token-level representation learning and the another for character-level representation learning. The BiLSTMs work as unary feature functions, whereas a static transition matrix comes in for pairwise feature functions. Afterward, Habibi et al. [10] adopted the model of Lample et al. [9] for BioNER.
- Luo et al. [22] adopted BiLSTM-CRF for NER in chemistry domain and applied an attention mechanism to leverage document-level context information. They employ abbreviation embeddings using a specific external library to handle abbreviations that frequently appear in chemical entities’ naming. Their model also relies on a static matrix to retrieve all the label-label transition relations in CRF.
- Dang et al. [12] developed D3NER to utilize various linguistic information under BiLSTM-CRF. D3NER creates a token embedding by aggregating several embeddings: a pre-trained word embedding, an abbreviation embedding, a POS embedding, and a character-level token embedding. Similarly, a transition matrix solely plays the role of pairwise feature functions.
- Wang et al. [11] introduced a multi-task learning framework for BioNER. They trained a model using several biomedical corpora together to overcome a limited amount of annotated biomedical corpora. Their model also adopts BiLSTM-CRF with a transition matrix.
- Yoon et al. [14] proposed aggregation of multiple expert models. They named it CollaboNet, where each expert model is mapped to a BiLSTM-CRF and is trained with each distinct corpus. Likewise, each BiLSTM-CRF has a transition matrix, corresponding to pairwise feature functions.
- Peters et al. [13] introduced ELMo as a pre-trained model. ELMo provides contextualized word embeddings for various downstream tasks. They also trained the ELMo-enhanced BiLSTM-CRF for NER.
- Lee et al. [23] released BioBERT by training BERT [24] for the use in the *Bioinformatics* domain. Similarly to ELMo, as a pre-trained model, BioBERT provides contextualized word embeddings and thus can be applied to downstream tasks. BioBERT achieved the state-of-the-art performance in several BioNER corpora.

## DTranNER: architecture and method

**Fig. 1:**
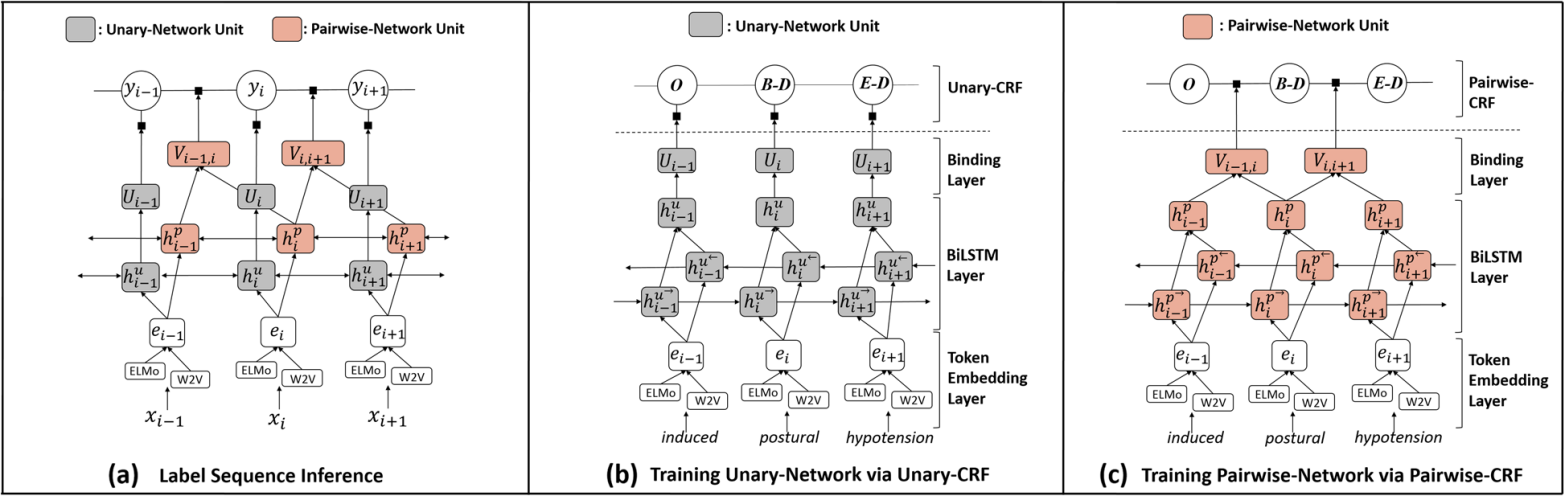
The overall architectures of the proposed framework DTranNER. **a** As a CRF-based framework, DTranNER is comprised of two separate, underlying deep learning-based networks: Unary-Network and Pairwise-Network are arranged to yield agreed label sequences in the prediction stage. The underlying DL-based networks of DTranNER are trained via two separate CRFs: Unary-CRF and Pairwise-CRF. **b** The architecture of Unary-CRF. It is dedicated to train Unary-Network. **c** The architecture of Pairwise-CRF. It is also committed to train Pairwise-Network. A token embedding layer is shared by Unary-Network and Pairwise-Network. A token-embedding is built upon by concatenating its traditional word embedding (denoted as “W2V”) and its contextualized token embedding (denoted as “ELMo”)

In this section, we present the proposed framework DTranNER as shown in Fig. 1. For parameter learning, the components (i.e., Unary-Network and Pairwise-Network) of DTranNER are systematically trained via two separate CRFs (i.e., Unary-CRF and Pairwise-CRF). Once trained, Unary-Network and Pairwise-Network are combined into a CRF for BioNER label sequence prediction. First of all, we describe how to build the token embeddings in our models. Although DTranNER is not limited to a specific DL architecture in the places of the underlying networks, from now on, we evaluate our framework using BiLSTM, which has been typically adopted in a majority of NER studies.

### Token-embedding layer

Given a sequence of *N* tokens (*x*_1_,*x*_2_,..., *x*_*N*_), they are converted token-by-token into a series of fixed-dimensional vectors (*e*_1_,*e*_2_,..., *e*_*N*_) via the token-embedding layer. Each token embedding is designed to encode several linguistic information of the corresponding token in the sentence. Each token embedding is thus built up by concatenating the traditional context-independent token embedding and its contextualized token embedding. These token embeddings are subsequently fed to Unary-Network and Pairwise-Network as the inputs. We do not consider additional character-level token embeddings unlike several models [9–12, 14, 20, 21], because ELMo [13] as our contextualized token embedding provider basically includes a character-level CNN model.

#### Context-independent token embedding

We use the pre-trained token vectors, *Wiki-PubMed-PMC*, created by Pyysalo et al. [25] to initialize the traditional token-embedding vectors. The pre-trained token vectors were made up by being trained on three different datasets: the abstracts of the PubMed database, the full-text articles of the PubMed Central (PMC) database, and the texts of a recent Wikipedia dump. It is available at [26]. We replace every out-of-vocabulary (OOV) token with a special *<UNK>* vector.

#### Contextualized token embedding

We employ ELMo [13] for the contextualized token embeddings. Unlike context-independent token embeddings based on GloVe [27] or Word2Vec [28], ELMo creates context-dependent token embeddings by reconsidering the syntax and semantics of each token under its sentence-level context. In particular, we adopt the in-domain ELMo model pre-trained on the PubMed corpus, which is available at [29].

### Unary-Network

As shown in Fig. 1b, Unary-Network takes token embeddings as inputs, put them into its own BiLSTM layer to extract task-specific contextual information in an ordered token-level sequence, and finally produces the *L*-dimensional score vectors as many as the number of tokens via its binding layer. The binding layer consists of two linear transformations with an activation function and a skip connection between them. That is, the binding layer is formulated as follows:

3 $$  {U_{i} = W_{2}^{u}(\sigma(W_{1}^{u}h_{i}^{u} + b_{1}^{u})+h_{i}^{u})+b_{2}^{u}},  $$

where *U*_*i*_ denotes the *L*-dimensional score vector exhibiting the suitability over all possible labels on the *i*th token, $h_{i}^{u}$ is the *i*-th hidden state from the BiLSTM layer, $W_{1}^{u}\in \mathbb {R}^{d\times d}$ and $W_{2}^{u}\in \mathbb {R}^{L\times d}$ are trainable weight matrices, and $b_{1}^{u}$ and $b_{2}^{u}$ are the bias vectors. Here, $W_{2}^{u}$ projects the *d*-dimensional vector obtained by both the feed-forward network and the skip connection to the *L*-dimensional output vector. We use an ELU as the activation function *σ*(·). As will be explained in the following section, Unary-Network is trained via the purpose-built CRF (i.e., Unary-CRF) for the parameter learning.

### Pairwise-Network

Pairwise-Network aims to extract contextual information related to pairwise labeling. This design explains why two consecutive hidden state vectors of the BiLSTM are involved in describing an edge connection in the CRF layer as shown in Fig. 1c. Pairwise-Network therefore generates *L*^2^-dimensional score vectors to match the number of possible label pairs on two tokens. We employ a bilinear model-based method [30] to exploit interactive features of two neighboring hidden state vectors. This method approximates a classical three-dimensional tensor with three two-dimensional tensors, significantly reducing the number of parameters. It is shown in the following equation:

4 $$  f_{i-1,i} = H(Q_{1}h_{i-1}^{p}\circ Q_{2}h_{i}^{p}),  $$

where *f*_*i*−1,*i*_ denotes the *m*-dimensional vector via the bilinear model of two neighboring hidden state vectors (i.e., $h_{i-1}^{p}$ and $h_{i}^{p}$) of the underlying BiLSTM layer; $Q_{1}\in \mathbb {R}^{c\times d}, Q_{2}\in \mathbb {R}^{c\times d}$, and $H\in \mathbb {R}^{m\times c}$ are trainable matrices; and ∘ denotes Hadamard product (i.e., element-wise product of two vectors). The binding layer has a skip connection as in Unary-Network. It is thus formulated as the following equation:

5 $$  V_{i-1,i} = W_{2}^{p}(\sigma(W_{1}^{p}f_{i-1,i} + b_{1}^{p}) + f_{i-1,i}) + b_{2}^{p},  $$

where $V_{i-1,i}\in \mathbb {R}^{L^{2}}$ denotes the score vector indicating the confidence values over all label combinations on the neighboring (*i*−1)th and *i*th tokens, $W_{1}^{p}\in \mathbb {R}^{m\times m}$ and $W_{2}^{p}\in \mathbb {R}^{L^{2}\times m}$ are trainable weight matrices, $b_{1}^{p}$ and $b_{2}^{p}$ are the bias terms, and *σ*(·) is an ELU activation. Similarly to Unary-Network, Pairwise-Network is also trained via the purpose-built CRF (i.e., Pairwise-CRF) for the parameter learning.

### Model training

Here, we explain how to train DTranNER. In order to facilitate the parameter learning of the two underlying networks (i.e., Unary-Network and Pairwise-Network), we establish two separate linear-chain CRFs, which are referred as Unary-CRF (Fig. 1b) and Pairwise-CRF (Fig. 1c), by allocating the two types of DL-based networks (i.e., BiLSTMs in our case) to the two purpose-built CRFs, respectively. The reason is that, when both Unary-Network and Pairwise-Network coexist in a single CRF, as Smith et al. [31] and Sutton et al. [32] claimed that the existence of a few indicative features can swamp the parameter learning of other weaker features, either one of the two networks starts to hold a dominant position, causing the other network to deviate from its optimal parameter learning. Our solution enables each network to notice own prediction error during the parameter learning. We explain in detail the effect of our training strategy in the Additional file 1.

In this study, note that each of Unary- and Pairwise-CRFs is a sufficient label sequence predictor or learner; in the sense, the conditional likelihood *P*_*u*_ of Unary-CRF is formulated as in Eq. 2, and the conditional likelihood *P*_*p*_ of Pairwise-CRF given the input sequence **x** with the length *N* is formulated as the following equation:

6 $$  P_{p}(\mathbf{y}|\mathbf{x}) = \frac{1}{Z}\exp{(\sum\limits_{i=2}^{N}V_{i-1,i}(y_{i-1},y_{i}))},  $$

where $Z\,=\,\sum \nolimits _{\mathbf {y}}{\exp {(\sum \nolimits _{i=2}^{N}V_{i-1,i}(y_{i-1},y_{i})}}$ is the normalization constant.

Rather than individually training multiple CRFs offline as in [31, 32], Unary-CRF and Pairwise-CRF are jointly trained in our training strategy by maximizing their product—i.e., $\prod {P_{\mathit {v} \in \{\mathit {u},\mathit {p}\}}(\mathbf {y}_{v}|\mathbf {x})}$—of the two likelihoods of Unary-CRF and Pairwise-CRF. By equivalently converting the objective function into the negative log likelihood, the optimization problem is written as the following equation:

7 $$ \min_{\theta_{u}, \theta_{p}} \sum\limits_{e}{-\log(P_{u}(\mathbf{y}^{e}|\mathbf{x}^{e};\theta_{u})) -\log(P_{p}(\mathbf{y}^{e}|\mathbf{x}^{e};\theta_{p}))},  $$

where **x**^*e*^ and **y**^*e*^ denote the *e*th training sentence example and its ground-truth label sequence, and *θ*_*u*_ and *θ*_*p*_ denote the model parameters of Unary-CRF and Pairwise-CRF respectively.

### Prediction

We explain the detail on how to infer label sequences with the trained DTranNER. Once trained via the two separate CRFs, Unary-Network and Pairwise-Network are arranged into a CRF to yield an agreed label sequence in the prediction stage. Note that Unary-Network and Pairwise-Network have distinct focuses derived by different roles, leading to learn their own specific representations. We combine them by multiplying them as a product of models [33]. More specifically, all the components obtained through the aforementioned training process—Unary-Network, Pairwise-Network, and the transition matrix—are organized in a CRF, as shown in Fig. 1a. The combined model is formulated in terms of the probability for a label sequence **y** given an input sequence **x** via the following equation:

8 $$ \begin{aligned} P(\mathbf{y}|\mathbf{x})&=P_{u}(\mathbf{y}|\mathbf{x})\cdot P_{p}(\mathbf{y}|\mathbf{x})\\ &\propto\exp{(\sum\limits_{i=1}^{N}U_{i}(y_{i})+\sum\limits_{i=2}^{N}A_{i-1,i})}\cdot\exp{(\sum\limits_{i=2}^{N}V_{i-1,i}(y_{i-1},y_{i}))}\\ &=\exp{(\sum\limits_{i=1}^{N}U_{i}(y_{i})+\sum\limits_{i=2}^{N}V_{i-1,i}(y_{i-1},y_{i})+\sum\limits_{i=2}^{N}A_{i-1,i})}. \end{aligned}  $$

As a result, we obtain the most likely label sequence using the Viterbi decoding.

## Experimental setup

### Datasets

We conducted our experiments with five BioNER benchmark corpora: BC2GM, BC4CHEMD, BC5CDR-chemical, BC5CDR-disease, and NCBI-Disease, which are commonly used in the existing literature [11, 12, 14, 23].

Table 1 shows the overall description of the five benchmark BioNER corpora. They are publicly available and can be downloaded from [34]. The BioCreative II Gene Mention (**BC2GM**) task corpus [35] consists of 20,128 sentences from biomedical publication abstracts and is annotated for mentions of the names of proteins, genes, and related entities. The BioCreative IV Chemical and Drug (**BC4CHEMD**) task corpus [36] contains the annotations for chemical and drug mentions in 10,000 biomedical abstracts. The BioCreative V Chemical Disease Relation (**BC5CDR**) corpus [37] is composed of mentions of chemicals and diseases that appeared in 1,500 PubMed articles. The NCBI-Disease corpus (**NCBI-Disease**) [38] is composed of 793 PubMed abstracts annotated for disease mentions. The aforementioned corpora cover four major biomedical entity types: gene, protein, chemical, and disease.

**Table 1:** BioNER corpora in experiments

Datasets	Number of Sentences	Entity Types	Entity Counts	Max Entity Length	Average Entity Length
BC2GM [35]	20128	Gene/Protein	24583	26 tokens	2.44 tokens
BC4CHEMD [36]	87682	Chemical/Drug	84310	137 tokens	2.19 tokens
BC5CDR-Chemical [37]	13935	Chemical/Drug	15935	56 tokens	1.33 tokens
BC5CDR-Disease [37]	13935	Disease	12852	19 tokens	1.65 tokens
NCBI-Disease [38]	7284	Disease	6881	22 tokens	2.21 tokens

### Training setup

In model training, we added L2 regularization penalty to the loss (i.e., Eq. 7) with the decay factor of 1×10^−5^. The *Glorot* uniform initializer of Glorot and Bengio [39] is used for initializing our weight matrices, and the biases are initialized with 0. All the activation functions are ELU (exponential linear unit). We set the minibatch size of model training to ten examples across all experiments. Our models are differentiable; thereby, the CRF and its underlying neural networks can be jointly trained end-to-end by backpropagation. We use the *Adam* optimizer of [40] with the learning rate of 0.001. In the training process, we renormalize all gradients whenever the L2 norm of the gradients exceeds 5 in every minibatch update. We applied layer normalization [41] to the outputs of the token embedding layer, and also applied weight normalization [42] to all the weight matrices of the binding layers of Unary-Network and Pairwise-Network. We used Dropout [43] with keep probability 0.5 in both the binding layers. We established our models within at most 50 epochs for all the corpora.

### Evaluation metrics

We evaluated all the methods using the precision, recall, and F1 score on the test sets of all corpora. We defined each predicted entity as correct if and only if both the entity type and the boundary were exactly matched to the ground-truth annotation. We used the python version of the evaluation script designed for CoNLL-2000 Benchmark Task, which can be downloaded from [44]. To get reliable results, we repeated every test *five times* with different random initialization and report the arithmetic mean.

## Results

### Overall performance comparison

We compared DTranNER with five state-of-the-art methods: **(1)** Att-BiLSTM-CRF [22], **(2)** D3NER [12], **(3)** Collabonet [14], **(4)** the multi-task learning-based model of Wang et al. [11], and **(5)** BioBERT [23]. Note that all the models except BioBERT employ a CRF as their top layer and rely on a static transition matrix. The performance values in terms of the *precision*, *recall*, and *F1*-score over all the corpora are presented in Table 2. DTranNER outperformed the current state-of-the-art models on four of five corpora—BC2GM, BC4CHEMD, BC5CDR-Disease, and BC5CDR-Chemical—in terms of F1 scores.

**Table 2:** Performance values in terms of the *precision* (%), *recall* (%) and *F1*-score (%) for the state-of-the-art methods and the proposed model **DTranNER**

Corpus	BC2GM			BC4CHEMD			BC5CDR-Chemical			BC5CDR-Disease			NCBI-Disease		
	P	R	F1	P	R	F1	P	R	F1	P	R	F1	P	R	F1
Att-BiLSTM-CRF (2017)	-	-	-	**92.29**	90.01	91.14	93.49	91.68	92.57	-	-	-	-	-	-
D3NER (2018)	-	-	-	-	-	-	93.73	92.56	93.14	83.98	85.40	84.68	85.03	83.80	84.41
Collabonet (2018)	80.49	78.99	79.73	90.78	87.01	88.85	94.26	92.38	93.31	85.61	82.61	84.08	85.48	87.27	86.36
Wang et al. (2018)	82.10	79.42	80.74	91.30	87.53	89.37	93.56	92.48	93.03	84.14	85.76	84.95	85.86	86.42	86.14
BioBERT (2019)	**85.16**	83.65	84.40	92.23	90.61	91.41	93.27	93.61	93.44	85.86	87.27	86.56	**89.04**	**89.69**	**89.36**
**DTranNER**	84.21	**84.84**	**84.56**	91.94	**92.04**	**91.99**	**94.28**	**94.04**	**94.16**	**86.75**	**87.70**	**87.22**	88.21	89.04	88.62

*Note:* The highest performance in each corpus is highlighted in **Bold**. We quoted the published scores for the other models. For Wang et al. [11], we conducted additional experiments to obtain the performance scores for two corpora (i.e., BC5CDR-Chemical and BC5CDR-Disease) using the software on their open source repository [45]

DTranNER achieved a much higher F1 score with higher precision than the current best performer (94.16% vs. 93.44%) for BC5CDR-Chemical, where its NER process was confused owing to many abbreviations despite its shorter average entity length as shown in Table 1. Thus, the pairwise transition network of DTranNER is shown to be advantageous in discovering abbreviation-formed entities.

### Ablation studies

We investigated the effectiveness of main components of our proposed method DTranNER through ablation studies.

#### Impact of unary- and pairwise-Networks

To investigate the contribution of Unary- and Pairwise-Networks to DTranNER, we trained experimental models by deactivating each component (i.e., either Unary-Network or Pairwise-Network) in turn from DTranNER and then measured the performance of the variant models on three benchmark corpora: BC5CDR-Chemical, BC5CDR-Disease, and NCBI-Disease. The results are shown in Table 3.

**Table 3:** Impact of Unary-Network and Pairwise-Network in terms of the F1-score (%)

Settings	BC5CDR-Chemical	BC5CDR-Disease	NCBI-Disease
Unary-CRF	93.01	86.14	86.94
Pairwise-CRF	93.27	86.05	86.71
Unary+Pairwise ensemble	93.25	86.78	87.09
DTranNER	94.16	87.22	88.62

*Note:* “Unary-CRF” denotes a variant model excluding Pairwise-Network from DTranNER, “Pairwise-CRF” denotes a variant model excluding Unary-Network from DTranNER, and “Unary+Pairwise ensemble” is an ensemble model of “Unary-CRF” and “Pairwise-CRF.” In the ensemble model, “Unary-CRF” and “Pairwise-CRF” were independently trained, and they voted over the sequence predictions by their prediction scores

The removal of either Unary-Network or Pairwise-Network from DTranNER caused the overall performance degradation in all the corpora by up to 1.91 percent points. That is, this ablation study presents that the performance achievement of DTranNER is attributed to not only an individual component but also the mutual collaboration of Unary-Network and Pairwise-Network. The relative importance between the two networks was not very clear.

We also compared DTranNER with an ensemble model of Unary-CRF and Pairwise-CRF, denoted as “Unary+Pairwise ensemble,” which were separately trained. The sequence prediction of the ensemble model was decided by voting with their sequence output scores. As shown in Table 3, the performance improvement of the ensemble model was marginal in BC5CDR-Chemical and NCBI-Disease. More important, the ensemble model was much worse than DTranNER in all corpora. This result indicates that yielding agreed label sequences between the two networks, which have separate views, as in DTranNER is more effective than their ensemble via simple voting.

#### Impact of separate BiLSTM layers of Unary- and Pairwise networks

Unary-Network and Pairwise-Network have an independent underlying layer which learns its role-specific representations. We investigate the impact of the separate underlying layers in the peer networks. For this purpose, we additionally built a variant model of DTranNER, denoted as “DTranNER-shared,” that forced Unary-Network and Pairwise-Network to share the parameters of their BiLSTM layers. As shown in Table 4 for the comparison result, it turned out that Unary-Network and Pairwise-Network benefit from the exclusive underlying layer.

**Table 4:** Impact of separate BiLSTM layers in terms of the F1-score (%)

Settings	BC2GM	BC5CDR-Chemical	BC5CDR-Disease	NCBI-Disease
DTranNER-shared	83.69	93.57	86.75	88.01
DTranNER	84.56	94.16	87.22	88.62

*Note:* “DTranNER-shared” is a variant model that shares the BiLSTM layer in “Unary-Network” and “Pairwise-Network.”

#### Embedding layer

We here investigate the impact of each element in the token embedding layer of DTranNER. For this purpose, we built two variants of DTranNER: (1) a model (denoted as “W2V”) whose token embedding consists of only 200-dimensional pre-trained token embedding [26] and (2) another model (denoted as “ELMo”) whose token embedding is solely comprised of 1024-dimensional ELMo embedding, which is obtained from the ELMo model [29] pre-trained on the PubMed corpus. The comparison results are presented in Table 5. The context-dependent token embeddings via the ELMo model bring significant performance improvement on the four benchmark corpora, especially on NCBI-Disease. Nevertheless, the best performance is consistently achieved by the combination of the context-dependent ELMo embedding and the traditional context-independent embedding.

**Table 5:** Impact of each component in the token embedding composition in terms of the F1-score (%)

Settings	BC2GM	BC5CDR-Chemical	BC5CDR-Disease	NCBI-Disease
W2V	82.03	92.64	85.17	84.88
ELMo	83.41	93.78	86.76	88.27
ELMo + W2V(=DTranNER)	84.56	94.16	87.22	88.62

*Note:* “W2V” is a variant model of DTranNER whose embedding layer uses only traditional context-independent token vectors (i.e., *Wiki-PubMed-PMC* [25]), “ELMo” is another variant model of DTranNER whose embedding layer uses only ELMo, and “ELMo + W2V” is equivalent to DTranNER

### Case studies

To demonstrate the advantage of the DL-based label-label transition model, which is the main feature of DTranNER, we compared several example outcomes yielded by DTranNER and Unary-CRF as shown in Table 6. Note that Unary-CRF is not equipped with this main feature. In addition, the label sequence predictions of DTranNER in Table 6 coincide with the ground-truth annotations.

**Table 6:** Case study of the label sequence prediction performed by DTranNER and Unary-CRF

Diseases/Chemicals		
Case 1	Unary-CRF	to enable diagnosis of ureteric stones or obstruction in patients with HIV infection who receive indinavir theraphy
	DTranNER	to enable diagnosis of ureteric stones or obstruction in patients with HIV infection who receive indinavir theraphy
Case 2	Unary-CRF	The present study was designed to investigate whether nociceptin / orphanin FQ and nocistatin could modulate
		impairment of learning and memory induced by scopolamine
	DTranNER	The present study was designed to investigate whether nociceptin / orphanin FQ and nocistatin could modulate
		impairment of learning and memory induced by scopolamine
Case 3	Unary-CRF	We report the case of a female patient with rheumatoid arthritis who developed acute cytolytic hepatitis due to meloxicam
	DTranNER	We report the case of a female patient with rheumatoid arthritis who developed acute cytolytic hepatitis due to meloxicam
Case 4	Unary-CRF	Reduced nicotinamide adenine dinucleotide phosphate - diaphorase (NADPH - d) histochemistry was also employed to
	DTranNER	Reduced nicotinamide adenine dinucleotide phosphate - diaphorase (NADPH - d) histochemistry was also employed to
Genes/Proteins		
Case 5	Unary-CRF	The MIC90 of ABK against coagulase type IV strains was rather high, 12.5 micrograms/ml
	DTranNER	The MIC90 of ABK against coagulase type IV strains was rather high, 12.5 micrograms/ml
Case 6	Unary-CRF	subtle differences between individual subunits that lead to species - specific properties of RNA polymerase I transcription
	DTranNER	subtle differences between individual subunits that lead to species - specific properties of RNA polymerase I transcription
Case 7	Unary-CRF	The S. typhimurium aspartyl / asparaginyl beta - hydroxylase homologue (designated lpxO) was cloned into
	DTranNER	The S. typhimurium aspartyl / asparaginyl beta - hydroxylase homologue (designated lpxO) was cloned into

*Note*: Unary-CRF is the purpose-built model excluding Pairwise-Network from DTranNER. The named entities inferred by each model are underlined in sentences

For Case 1, Unary-CRF failed to detect one of the boundaries of the disease-type entity “ureteric stones or obstruction” because of the intervention of the inner conjunction “or,” while DTranNER precisely determined both boundaries. For Case 2, Unary-CRF failed to identify the chemical-type entities enumerated via the conjunctions “/” and “and,” whereas DTranNER exactly identified all the separate terms. For Case 3, Unary-CRF failed to determine the left boundary of the single-token entity “hepatitis” by mistakenly regarding “acute” and “cytolytic” as its constituent elements, whereas DTranNER exactly distinguished them from this entity by understanding the contextual relations. For Case 4, DTranNER correctly identified the two entities, where the latter is the abbreviation of the former, but Unary-CRF failed. For Case 5, Unary-CRF ignored the gene-type entity “coagulase type IV” by mistakenly regarding “type” and “IV” as generic terms, whereas DTranNER correctly identified it by reflecting the contextual correlations between its constituent elements. For Case 6, DTranNER correctly identified both boundaries of the gene-type entity “RNA polymerase I” by exploiting the contextual clues on the consecutive pairs, 〈“polymerase” and “I” 〉 and 〈“I” and “transcription” 〉, though “I” solely looks ambiguous; in contrast, Unary-CRF failed to determine the right boundary because it classified “I” as a generic term. For Case 7, DTranNER correctly extracted the lengthy entity by grasping the correlation between the neighboring tokens (i.e., “hydroxylase” and “homologue”), whereas Unary-CRF failed to handle this lengthy entity.

Summing up, DTranNER successfully supports various cases which would be very difficult without the contextual information, and these cases indeed show the benefit of DTranNER for BioNER.

## Conclusion

In this paper, we proposed a novel framework for BioNER, for which we call **DTranNER**. The main novelty lies in that DTranNER learns the label-label transition relations with deep learning in consideration of the context in an input sequence. DTranNER possesses two separate DL-based networks: Unary-Network and Pairwise-Network; the former focuses on individual labeling, while the latter is dedicated to assess the transition suitability between labels. Once established via our training strategy, these networks are integrated into the CRF of DTranNER to yield agreed label sequences in the prediction step. In other words, DTranNER creates the synergy leveraging different knowledge obtained from the two underlying DL-based networks. As a result, DTranNER outperformed the best existing model in terms of the F1-score on four of five popular benchmark corpora. We are extending DTranNER to utilize unlabeled biomedical data. This extension is meaningful in several aspects: (1) building a more-generalized model using a wide range of biomedical literature, (2) rapidly incorporating up-to-date biomedical literature by skipping time-consuming annotation, and (3) reducing annotation cost.

## Supplementary information

**Additional file 1** Model training strategy in the proposed framework. The effect of the proposed model training strategy is not shown in the main manuscript. The learning curves during model training demonstrate the reason why the training strategy facilitates the parameter learning of the underlying deep learning-based models under our proposed framework.

## Data Availability

The code for our models and instructions for the use can be found on GitHub https://github.com/kaist-dmlab/BioNER. The datasets used for performance evaluation and analysis during the current study are available in the MTL-Bioinformatics-2016 repository, https://github.com/cambridgeltl/MTL-Bioinformatics-2016.

## Abbreviations

BiLSTM: Bidirectional long short-term memory
BioNER: Biomedical named entity recognition
CNN: Convolutional neural network
CRF: Conditional random field
DL: Deep learning
NER: Named entity recognition
NLP: Natural language processing
POS: Part-of-speech
