# Supplementary material for: DTranNER: biomedical named entity recognition with deep learning-based label-label transition model
Source: BMC Bioinformatics. 2020 Feb 11;21:53. doi: 10.1186/s12859-020-3393-1 (PMC7014657; doi:10.1186/s12859-020-3393-1)
Supplement: Supplementary file 1 — Additional file 1 Model training strategy in the proposed framework. The effect of the proposed model training strategy is not shown in the main manuscript. The learning curves during model training demonstrate the reason why the training strategy facilitates the parameter learning of the underlying deep learning-based models under our proposed framework. [file 12859_2020_3393_MOESM1_ESM.pdf]

Additional File 1: Proposed Training Strategy in DTranNER

May 31, 2019

Proposed Training Strategy

We investigate the effect of our proposed training strategy where Unary-Network and Pairwise-Network are successfully trained by being partitioned into the two separate, training-purposed CRFs: Unary-CRF and Pairwise-CRF. We highlight the effectiveness of the solution by comparative evaluation; thus, we additionally built an experimentally-purposed model where Unary-Network and Pairwise-Network are placed together on a linear-chain CRF during model training. We broke down the training errors monitored by the two different training ways on the four biomedical corpora: BC2GM, BC4CHEMD, BC5CDR, and NCBI-Disease, as shown in Figure 1. Note that the y-axis of the learning curves in those graphs indicates the corresponding negative log likelihood loss, while the x-axis is the number of epochs in the graphs. In Figure 1 (a), it is observed that all Pairwise-CRFs are far from being converged during parameter learning. In other words, they exhibit so-called **"under-fitting"** phenomenon. In contrast, Unary-CRFs and Pairwise-CRFs of DTranNER, as shown in Figure 1 (b), continuously seek to minimize own training loss as we expected. These experimental analysis presents that the proposed training strategy is essential in the parameter learning of the underlying deep learning-based models of DTranNER.

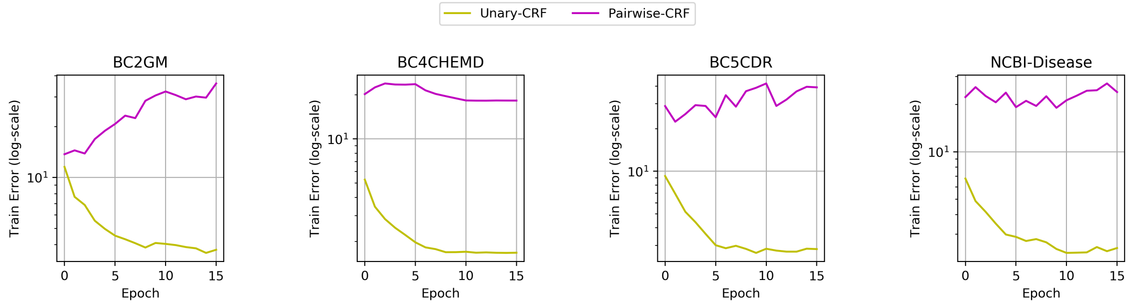

(a) The learning curves of the models where Unary-Network and Pairwise-Network are placed in a linear-chain CRF.

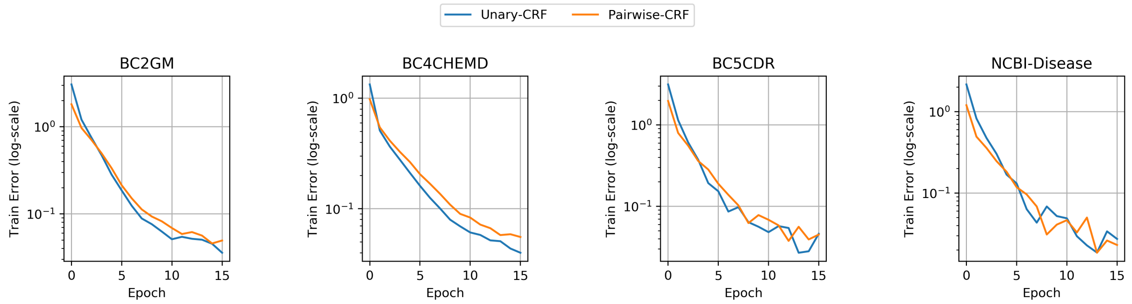

(b) The learning curves of the models monitored via the proposed training strategy.

Figure 1: The learning curves were monitored by two different training strategies. Figure 1 (a) exhibits "under-fitting" phenomenon. Figure 1 (b) exhibits the full convergence.
